# Intimate partner violence, HIV and sexually transmitted infections in fishing, trading and agrarian communities in Rakai, Uganda

**DOI:** 10.1186/s12889-019-6909-8

**Published:** 2019-05-17

**Authors:** Bushra Sabri, Andrea L. Wirtz, Joseph Ssekasanvu, Bareng A. S. Nonyane, Fred Nalugoda, Joseph Kagaayi, Robert Ssekubugu, Jennifer A. Wagman

**Affiliations:** 1 School of Nursing, Johns Hopkins University, 525 North Wolfe Street, Room 456, Baltimore, MD-21205 USA; 2 Johns Hopkins Bloomberg School of Public Health, Johns Hopkins University, Baltimore, MD-21205 USA; 3 Rakai Health Sciences Program, Uganda Virus Research Institute, Nakiwogo Road, P.O. Box 49, Entebbe, Uganda; 4 Division of Global Public Health, University of California, San Diego, USA

**Keywords:** Intimate partner violence, HIV, Sexually transmitted infections

## Abstract

**Background:**

Intimate partner violence (IPV), HIV and sexually transmitted infections (STI) can contribute to disparities in population health, depending on the individual, social and environmental factors characterizing a setting. To better understand the place-based determinants and patterns of these key interrelated public health problems in Uganda, we compared risk factors for IPV, HIV and STI in fishing, trading and agrarian communities in Rakai, Uganda by gender.

**Method:**

This study used cross-sectional data collected from 14,464 sexually active men (*n* = 6531) and women (*n* = 7933) as part of the Rakai Community Cohort Study, a population-based open cohort study of men and women aged 15–49 years. We used multilevel modified poisson regression models, which incorporated random intercepts for community and households. Factors associated with IPV, HIV and STI were assessed separately for men and women in fishing, trading and agrarian communities.

**Results:**

A larger proportion of participants in the fishing communities than those in trading and agrarian communities were HIV positive, engaged in HIV risk behaviors, had STI symptoms and reported perpetration of or victimization by IPV. Female gender was a shared correlate of IPV, HIV and STI in the fishing communities. Engagement in multiple sexual relationships or partner’s engagement in multiple relationships were shared correlates of IPV, and HIV in agrarian communities and IPV and STI in trading communities.

**Conclusion:**

Programs should target factors at multiple levels to reduce risk for syndemic conditions of HIV, STI and IPV in Rakai, Uganda particularly among men and women in fishing communities.

## Background

Intimate partner violence (IPV) and HIV infection are key interrelated public health problems worldwide, disproportionately impacting sub-Saharan Africa. Globally, 30% of ever-partnered women experience lifetime prevalence of IPV, with slightly higher estimates (36.6%) reported in sub-Saharan Africa [1]. The burden of HIV is also disproportionately high in this region with an estimated 25.6 million people living with HIV in 2016, compared to global estimates of 36.7 million [2]. A complex relationship exists between IPV and HIV infection [3–10] with multiple pathways and bi-directionality between the two outcomes [4]. Sexual IPV may directly lead to HIV acquisition by forced condomless sex or inability to negotiate condom use with an infected, violent partner. Further evidence suggests that men who use violence against their partners are more likely to engage in a range of high risk sexual (e.g., multiple partners and condomless sex) and/or drug use (e.g., hazardous alcohol use and injection drug use [11]) behaviors, relative to men who are non-violent [9]. Biological mechanisms are also thought to play a role by indirectly increasing risk for HIV and other sexually transmitted infections (STIs) through trauma to the vaginal or rectal mucosa [7]. Further, chronic stress experienced by IPV victims is hypothesized to increase susceptibility to HIV/STI infection by compromising the immune system [9]. Conversely, disclosure of one’s HIV status to a partner can also act as an antecedent to perpetration of IPV.

The co-occurring and intersecting nature of the epidemics of IPV, HIV and STI are referred to as syndemic, or a set of two or more epidemics, interacting synergistically and contributing to excess burden of disease in a population [13]. The presence of syndemic conditions are known to be driven by multiple factors (at different ecological levels, such as individual, relationship and community) in the social environment. The syndemic theory provides a useful framework for studying the interaction between co-occurring epidemics and identifying contextual factors that could be targeted in addressing these epidemics [13, 14]This paper uses a syndemic approach [13] to examine prevalence and correlates of IPV, HIV and STI among men and women in three distinct community types in Rakai, Uganda.

Substantial evidence has already been generated by the Rakai Health Sciences Program (RHSP) on the relationship between IPV and HIV infection in agrarian setting of Rakai, Uganda. Data collected over a period of 10 years from 15,080 adult women found 59 and 29% experienced lifetime, and past year IPV (including physical, sexual and verbal), respectively [5, 12]. In an analysis of Rakai data, 14.4% of adolescent females (15–19 years) experienced forced first sex [12]. Many adverse reproductive and relationship-level outcomes have been associated with partner-level violence, including unintended pregnancy [5], higher rates of genital tract symptom [5], attempted abortion [15], and union dissolution through divorce or separation [16]. Significant associations were also found between IPV and incident HIV. The estimated adjusted population fraction of HIV attributable to IPV in Rakai is 22% (95% CI 12.5–30.4) [5]. Longitudinal data from Rakai, Uganda, has demonstrated that IPV is a risk factor for HIV incidence, with an adjusted HIV incidence rate ratio (IRR) of 1.55 (95% CI: 1.25–1.94) among women with lifetime experiences of IPV relative to those with no history of IPV [5]. Meta-analyses of these data from Uganda, as well as South Africa and Rwanda, demonstrate similar associations with sexual IPV (pooled odds ratio (OR): 1.77, 95% CI: 1.00–1.46), physical IPV (pooled OR: 1.22; 95% CI: 1.02–1.46), and any IPV with HIV acquisition (pooled OR:1.28; 95%CI:1.00–1.64) [6]. Risk for HIV acquisition associated with IPV may be compounded by the presence of other common risk factors, such as co-infection with STIs, including genital ulcerative diseases [17–20]. However, the impact of these synergistic interactions on IPV has not been fully explored across or between all age groups in the different community types in Rakai. Shared risk factors contributing to both incident IPV and HIV include gender inequality, young age, lower socio-economic status, unemployment and poverty [7, 15]. Taken together, these results suggest an urgent need for combination programming to prevent violence against women and HIV infection.

To be effective, prevention programming must be tailored to meet the needs of those exposed to the intervention. Global research has found different forms of IPV (e.g., sexual IPV vs. physical IPV) occur at different rates depending on the type of geographic location characterizing the population [21]. Findings from Nigeria, for instance, suggest an increased burden of IPV in agrarian communities, relative to urban communities [22]. Systematic reviews to understand the contribution of living in urban or rural communities on IPV has further identified differences in frequency, duration and severity of IPV perpetration [21]. Little research, however, has investigated environmental differences in the relationship between IPV and STIs, including HIV, in the distinct community types found in rural African settings like Rakai. As proven prevention approaches are extended to new regions throughout Rakai and neighboring districts (and across Uganda and sub-Saharan Africa) it is important to understand the geographic places, populations and characteristics of those most affected by HIV/STIs and IPV to tailor programs that can effectively meet the specific needs of those in each area. In this study, we analyzed data from the Rakai Community Cohort Study (RCCS), an ongoing, longitudinal HIV and reproductive health surveillance cohort to assess the epidemiology of IPV victimization and perpetration among women and men across distinct community types, including fishing, trading and agrarian communities, in Rakai Uganda. Employing a syndemic [13, 14] approach, this study aimed to identify unique correlates of IPV, HIV, and STI outcomes in these communities.

## Methods

We analyzed cross-sectional data collected between January 2010 and June 2011 as part of the RCCS, a population-based open cohort study of consenting men and women aged 15–49 years. The analysis was restricted to data collected from lifetime sexually active male and female RCCS participants living (for at least 6 months) in 4 fishing, 15 trading and 19 agrarian communities in Rakai, Uganda. The RCCS was initiated in 1994 and between 2010 and 2011 (when data for the current analysis were collected), the survey was conducted in 38 communities in the Rakai district of southcentral Uganda [23]. Each survey round was preceded by a household census of all residents. During each survey round, a standardized questionnaire covering self-reported behaviors was administered privately by interviewers of the same sex, and biological samples were collected to test for HIV and other relates issues. All RCCS respondents provide written informed consent. The study was approved by Ugandan and US Institutional Review Boards (The Uganda Virus Research Institute’s Research and Ethics Committee, the Uganda National Council for Science and Technology, and the Western IRB).

### Measures

Dependent variables of interest included HIV, STI symptomatology, and IPV victimization or perpetration. ***HIV infection*** was diagnosed using a three-rapid test algorithm and confirmed by two enzyme immunoassays (EIAs) with Western blot or PCR confirmation of discordant EIAs and/or new seroconverters. ***STI symptoms*** were self-reported and included genital ulcers, genital discharge or dysuria in the past 12 months. ***IPV victimization*** of women was assessed by women’s reports of physical or sexual abuse by an intimate partner within the past 12 months. Recent physical and sexual IPV were measured using an adapted version of the Conflict Tactics Scale [24]. Physical IPV was measured by asking if within the past 12 months, the current partner engaged in behaviors such as pushing, pulling, grabbing, kicking, slapping, punching, burning and strangling. Sexual IPV was measured by asking if within the past 12 months the current partner engaged in sexually abusive behaviors such as physically forcing a partner to have sex or forcing to perform sexual activities. ***IPV perpetration*** by males was assessed by asking the same questions (described above) about physical or sexual abuse, but in the opposite direction, e.g., if within the past 12 months, the partner pushed, pulled or slapped his partner.

#### Individual background characteristics

Age was categorized into 15–24, 25–34, and 35+ years. The categories of marital status included the following: never married, married-polygamous, married-monogamous and previously married (i.e., divorced, separated or widowed). Religion included Catholic, Protestant, Muslim and other. Education was the number of years of education. Occupation was categorized as agriculture, housework, fishing, shopkeeper, business or trading, bar or restaurant worker and other. Household characteristics included household wealth categorized into high, middle and low based on building material, modern objects and utilities of the household. Family size was based on number of individuals in each family. We also assessed for forced sex at sexual debut (dichotomous) and age of first sexual intercourse. Age of first sexual intercourse was categorized as 12 years and under, adolescence (13–17 years), and 18 years or older.

***Sexual behaviors*** included dichotomous variables of participant’s alcohol use before sex in the past 12 months (yes/no), partner’s alcohol use before sex in the past 12 months (yes/no), and perceived likelihood of the partner being exposed to HIV (Very likely/somewhat likely and not likely). We assessed the age gap between partners using the following categories: 1–5 years, 6–10 years, 11–15 years, and more than 15 years. Condom use was categorized as always, sometimes or never used condoms in the past year with a non-primary partner or a non-marital partner. Individuals with multiple sexual partners were defined as those who had two or more sexual partners (marital or non-marital) within the past year. Partner’s multiple sexual relationships was defined as a respondent’s report that their partner had sexual relations with persons other than themselves in the past 12 months. Recent casual partnerships were assessed using self-report categorized into two: a) recent partner being a spouse or a consensual regular partner, and b) recent partner being an irregular partner. Numbers of partners outside the community or residence were coded as none, one, or two or more partners outside community of residence. All sexual behavior variables were assessed by self-report.

#### Social/community norms

Justification of wife beating was assessed by asking “do you think a man is justified in beating his wife or partner if she neglects household responsibilities, disobeys her husband or elders, uses contraception without permission of her husband, refuses to have sex with her husband/partner, if her husband/partner learns about her positive HIV sero status, if her husband/partner learns about his HIV own sero status, if she argues over money, if she is unfaithful or other reasons.

#### Community types

Fishing community includes communities on Lake Victoria whose primary occupation relates to fishing (e.g., fish processing, boat ownership) [27]. Trading communities are classified based on the proportion of population reporting trading as their primary occupation being in the top quartile among all RCCS communities [27]. Additional characteristics include households in big town, many households, high mobility, and easy access to major roads. Agrarian communities include those with primary occupation as agriculture. These communities are far off from the main roads, and from big or small towns. Agrarian communities are also characterized by very limited trading activities.

### Statistical analysis

Descriptive analysis with chi-square tests was conducted to compare the three community types based on IPV victimization/perpetration, HIV infection, STI symptoms and socio-demographic/household characteristics. Multivariable regression models were run for the total population, followed by separate multivariable models which were implemented in stratified form by gender and community type. This allowed for identification of individual-level, relationship-level and household-level correlates of IPV, HIV infection and STI symptoms across gender and community types. Variables significantly related with the outcomes at *p* < 0.05 in bivariate analysis were included in the multivariable model. We assessed for multicollinearity using the correlation coefficient matrix. Multilevel modified Poisson regression models with robust variance were used to estimate adjusted prevalence ratios (adjPRR) and 95% confidence intervals (95% CI). IPV was included in all models for the HIV and STI outcomes. Multivariate models were fit for the whole population and stratified by gender for the three community types. Models were also stratified by occupation. We included a random intercept for community and households. Since the fishing community had only 4 sub-communities, we included community as a fixed effect variable and household as a random effect variable for the fishing communities. For trading and agrarian communities both community and households were included as random effects. All analyses were performed with Stata Version 14.0 (College Stations, TX) using the *gllamm* procedure.

## Results

The sample consisted of 14,464 sexually active respondents (54.8% females, *n* = 7933; 45.1% males, *n* = 6531). Approximately 23% of the participants (*n* = 3316) were living with HIV (women 25.6% vs. men 19.5%, *p* < 0.001) and 20.8% (*n* = 2378) self-reported STI symptoms (women 26.1% vs. men 15.6%, *p* < 0.001). Recent IPV victimization was reported by 21.2% (*n* = 1535) of women while 11.8% (*n* = 715) of men reported perpetration.

### Community and gender differences in HIV infection, past year STI symptoms, IPV experiences and HIV/STI risk behaviors: descriptive findings

As shown in Table 1, sexually active participants in the fishing communities were significantly more likely to be HIV positive (42.3%) compared to trading (16.7%) and agrarian communities (15.6%, *p* < 0.001). A greater proportion of participants in the fishing communities also reported STI symptoms (34.3% versus 15.9% in trading and 16.3% in agrarian communities; *p* < 0.001) in the past year (Table 1). Table 2 shows that fishing communities were also characterized by a higher proportion of men reporting perpetration of IPV in the past year (17.5%), relative to men in the trading (9.2%) and agrarian (9.4%) communities. Similarly, as shown in Table 2, a higher proportion of women in the fishing communities (33.4%), relative to women in the trading (17.9%) and agrarian (17.1%) communities reported past year IPV victimization. Compared to agrarian (13.8%, *n* = 250), and trading communities (14.5%, *n* = 251), more than a quarter of the women in the fishing communities also reported forced sex experiences during their first sexual encounters (26.8%, *n* = 470, Table 2). With respect to HIV-related risk behaviors (Table 1), a significantly greater proportion of the participants in the fishing communities than those in trading and agrarian communities reported having multiple sexual partners (44.1%, *n* = 1570), recent casual relationships (5.9%, *n* = 222), two or more partners outside the community in the past 12 months (10.7%, *n* = 379), having a partner with multiple sexual relationships (57.1%, *n* = 765), use of alcohol before sex (39.7%, *n* = 1502) and partner’s use of alcohol before sex (40.6%, *n* = 1537) (*p* < 0.001; Table 1). Further, a significantly higher proportion of participants in the fishing communities perceived their partner to be exposed to HIV (82.1%, *n* = 3075) (*p* < 0.001). Among the household variables, a majority of participants in the fishing communities had low household wealth (65.2%, *n* = 2403) when compared to those in trading (10.3%, *n* = 499) and agrarian communities (12.4%, *n* = 728) (*p* < 0.001; Table 1).

**Table 1:** Community-level differences in HIV, STI, IPV and HIV/STI risk behaviors

	Fishing	Trading	Agrarian	*P* value
*Age*				
15 to 24 years	27.06 (1049)	36.72 (1989)	37.40 (2484)	< 0.001
25 to 34 years	45.30 (1756)	36.28 (1965)	34.25 (2275)	
35 years and above	27.63 (1071)	26.99 (1462)	28.35 (1883)	
*Gender*				
Males	51.7 (1959)	41.7 (2013)	43.7 (2559)	< 0.001
Females	48.3 (1827)	58.3 (2814)	56.3 (3292)	
*Education*				
No education	9.75 (378)	3.31 (179)	4.02 (267)	< 0.001
Primary education	72.39 (2806)	56.42 (3055)	63.15 (4193)	
Secondary education	15.94 (618)	29.60 (1603)	27.38 (1818)	
High school and above	0.77 (30)	3.29 (178)	1.05 (70)	
Vocational/Professional	1.14 (44)	7.39 (400)	4.40 (292)	
*HIV Status*				
Positive	42.3 (1599)	16.7 (806)	15.6 (911)	< 0.001
Negative	57.7 (2184)	83.3 (4012)	84.4 (4930)	
*STI*	34.3 (996)	15.9 (613)	16.3 (769)	< 0.001
*Household Wealth*				
High	25.49 (962)	70.96 (3839)	50.37 (3343)	< 0.001
Middle	9.49 (358)	18.52 (1002)	37.22 (2470)	
Low	65.02 (2454)	10.52 (569)	12.42 (824)	
*Any Physical or Sexual IPV (Past year)*	25.1 (894)	14.3 (624)	13.7 (731)	< 0.001
*Condom use in past year*				
Never used condom	39.0 (772)	40.6 (694)	41.5 (881)	< 0.001
Sometimes used condom	36.5 (722)	30.3 (518)	30.4 (646)	
Always used condom	24.5 (485)	29.1 (498)	28.1 (598)	
*Multiple sexual partners: Past 12 months*	44.1 (1570)	21.1 (910)	21.6 (1138)	< 0.001
*Recent partner was a casual partner*	5.87 (222)	2.48 (113)	2.52 (139)	< 0.001
*Partner’s multiple sexual relationships: Past year*	57.1 (765)	52.9 (861)	46.5 (940)	< 0.001
*Alcohol use before sex*	39.7 (1502)	23.7 (1078)	26.8 (1479)	< 0.001
*Partner’s alcohol use before sex*	40.6 (1537)	28.8 (1313)	32.6 (1796)	< 0.001
*Perceived likelihood of partner being exposed to HIV*				
-Very likely or somewhat likely	82.1 (3075)	74.1 (3366)	74.3 (4088)	< 0.001
*Number of partners outside the community: Past 12 months*				
One partner	24.5 (867)	26.2 (1129)	22.5 (1182)	< 0.001
Two or more partners	10.7 (379)	5.34 (230)	5.17 (272)

**Table 2:** Gender differences in HIV/STI, IPV, and HIV risk behaviors in the three community types

	Fishing		Trading		Agrarian	
	Men*	Women*	Men*	Women*	Men*	Women*
HIV	35.4 (692)	49.7 (907)	12.9 (260)	19.4 (546)	12.5 (320)	17.9 (591)
STI	27.7 (467)	43.5 (529)	10.0 (183)	21.4 (430)	11.1 (259)	21.2 (510)
Physical or Sexual IPV in the past year	17.5 (326)	33.4 (568)	9.16 (168)	17.9 (456)	9.40 (221)	17.1 (510)
Perpetration (men)						
Victimization (women)						
*Condom use in past year*						
Never used condom	36.0 (465)	44.6 (307)	33.1 (301)	49.1 (393)	35.7 (445)	49.6 (436)
Sometimes used condom	36.6 (472)	36.3 (250)	30.5 (277)	30.1 (241)	31.7 (395)	28.5 (251)
Always used condom	27.4 (354)	19.0 (131)	36.4 (331)	20.8 (167)	32.6 (406)	21.8 (192)
*Multiple sexual partners Past Year (Two or more)*	64.8 (1202)	21.6 (368)	38.9 (708)	8.07 (202)	40.7 (950)	6.41 (188)
*Two or more sex partners outside the community* *Past Year*	17.8 (327)	3.06 (52)	9.61 (174)	2.24 (56)	9.80 (228)	1.50 (44)
*Partner’s multiple sexual relationships* Past Year	30.7 (135)	70.1 (630)	9.95 (38)	66.2 (823)	11.1 (60)	59.4 (880)
*Recent casual relationship*	10.7 (209)	0.71 (13)	4.69 (90)	0.87 (23)	4.69 (115)	0.78 (24)
*Alcohol use before sex*	47.5 (930)	31.3 (572)	33.8 (649)	16.3 (429)	38.5 (943)	17.5 (536)
*Partner’s alcohol use before sex*	33.4 (654)	48.4 (883)	17.1 (329)	37.4 (984)	18.8 (462)	43.5 (1334)
*Force was used when had sex for the first time*	0	26.83 (470)	0	14.51 (251)	1 (100)	13.38 (250)

*All values for gender differences within communities were significant at *p* < 0.001

### Factors associated with HIV infection, past year STI symptoms and IPV experiences, by community type and gender: multivariate findings

#### HIV Outcome

##### Fishing Communities

In fishing communities, participants in the older age groups (25–34 years: *adjPRR* = 1.80, 95% *CI* = 1.22–2.65 and 35 years and above: *adjPRR* = 1.87, *95%* CI = 1.25–2.78) were significantly more likely to be HIV positive than those in the younger age groups (15–24 years of age). Men were less likely to be living with HIV than women (*adjPRR* = 0.57, *95% CI* = 0.42–0.78). Having a partner who was HIV infected was associated with being HIV positive (*adjPRR* = 1.70, *95% CI* = 1.04–2.76).

Stratified analysis by gender and community type (Table 3) demonstrated that men and women in the older age groups in fishing communities were more likely to be HIV positive than those in the younger age groups. Notable differences across genders were observed in household characteristics. Men in fishing communities who belonged to larger families were more likely to be HIV positive than those who belonged to smaller families (*adjPRR* = 1.07, *95% CI* = 1.01–1.14). In contrast, for women, a larger family size was related to a lower likelihood of being HIV positive (*adjPRR* = 0.94, *95% CI* = 0.90–0.97). While household wealth was not significantly associated with HIV status among men, women in fishing communities who belonged to low income/wealth households were significantly more likely to be HIV positive than those who belonged to high income households (*adjPRR* = 0.94, *95% CI* = 0.90–0.97). After adjusting for other variables, sexual behaviors were not significantly correlated with a positive HIV status among men. However, for women, use of alcohol before sex (*adjPRR* = 1.17, *95% CI* = 1.01–1.36) and belief that the male partner was likely to be exposed to HIV (*adjPRR* = 1.79, *95% CI* = 1.30–2.46) were significantly associated with HIV infection.

**Table 3:** HIV status as an outcome: IPV and other factors related to HIV status among males and females in the three community types

	Males			Females		
	Fishing	Trading	Agrarian	Fishing	Trading	Agrarian
Individual characteristics						
*Age*						
15 to 24 years (Ref)			–	–	–	–
25 to 34 years	**1.91 (1.19–3.05)**	**2.29 (1.47–3.57)**	**1.60 (1.09–2.34)**	**1.47 (1.19–1.82**	0.79 (0.52–1.21)	**1.49 (1.01–2.19)**
35 years and above	**1.86 (1.08–3.22)**	**4.26 (2.72–6.65)**	**2.37 (1.61–3.48)**	**1.58 (1.24–2.00)**	1.07 (0.67–1.73)	1.25 (0.82–1.89)
*Marital Status*						
Never married (Ref)	–	–	–	–	–	–
Married with multiple partners	1.09 (0.64–1.84)	2.88 (1.53–5.42)	**2.06 (1.11–3.81)**	1.26 (0.75–2.12)	2.97 (0.91–9.69)	2.28 (0.56–9.32)
Married- monogamous	1.10 (0.57–2.11)	**3.50 (2.02–6.06)**	**2.44 (1.42–4.18)**	1.40 (0.84–2.33)	**3.57 (1.16–11.05)**	2.85 (0.66–12.30)
Previously married	1.44 (0.92–2.27)	**3.81 (2.15–6.75)**	**2.76 (1.49–5.10)**	1.46 (0.87–2.44)	**3.28 (1.63–6.63)**	**4.23 (1.07–16.6)**
*Religion*						
Catholic	–	–	–	–	–	–
Protestant	1.29 (0.94–1.77)	**0.66 (0.44–0.98)**	0.96 (0.72–1.27)			
Muslim	0.82 (0.53–1.25)	**0.52 (0.35–0.78)**	**0.61 (0.40–0.94)**			
Other or None	0.79 (0.42–1.46)	0.56 (0.27–1.25)	1.27 (0.78–2.07)			
*Education (Years)*		0.92 (0.83–1.00)	–	–	0.98 (0.84–1.15)	–
*Type of Occupation*						
Fishing	Ref	–	–	–	–	–
Housework	–	–	–	Ref	Ref	Ref
Agriculture	0.18 (0.02–1.30)	1.19 (0.84–1.68)	Ref	0.73 (0.51–1.04)	0.83 (0.47–1.45)	2.20 (0.75–6.48)
Business/Trading	0.63 (0.39–1.01)	Ref	1.34 (0.92–1.96)	0.96 (0.78–1.18)	0.75 (0.38–1.51)	2.39 (0.91–6.29)
Bar/Restaurant	–	–	–	0.88 (0.71–1.08)	1.21 (0.61–2.42)	**2.92 (1.15–7.43)**
Other	**0.62 (0.43–0.91)**	0.96 (0.67–1.36_	1.12 (0.92–1.36)	0.87 (0.71–1.08)	0.55 (0.27–1.11)	1.79 (0.70–4.56)
*Age of first sexual intercourse*			–			
18 and older (Ref)	–	–		–	–	–
Childhood (12 years and under)				1.30 (0.92–1.84)	0.86 (0.62–1.20)	1.85 (0.75–4.57)
Adolescence (13–17 years)				1.11 (0.91–1.34)	1.37 (0.68–2.74)	1.50 (0.92–2.46)
*Forced first sex*	–	–	–	1.05 (0.91–1.22)	0.49 (0.06–3.56)	1.14 (0.79–1.65)
Relationship charactersitics						
*Any Physical or Sexual IPV*	1.00 (0.71–1.41)	0.86 (0.53–1.38)	1.38 (0.92–2.10)	1.07 (0.93–1.22)	1.27 (0.76–2.11)	0.91 (0.62–1.32)
*Age Gap Between Partners*						
1–5 years’ gap (Ref)	–	–	–		–	
6–10 years’ gap (2)	1.23 (0.92–1.64)	0.96 0.76–1.21)	0.95 (0.70–1.31)	–	0.86 (0.62–1.21)	–
11 or more years (3)	1.26 (0.83–1.90)	0.63 (0.39–1.02)	0.86 (0.59–1.25)		1.37 (0.68–2.74)	
*Condom use in past year*						
Never used condom (Ref)						
Sometimes used condom	–	–		–	1.20 (0.94–1.54)	–
Always used condom					1.32 (0.69–2.50)	
*Multiple sexual partners: Past 12 months*	1.18 (0.84–1.66)	1.37 (0.95–1.97)	**1.38 (1.15–1.65)**	1.05 (0.89–1.23)	1.16 (0.75–1.80)	**1.44 (1.03–2.02)**
*Partner’s multiple sexual relationships*	–	–	–	1.15 (0.96–1.37)	1.38 (0.62–3.07)	1.26 (0.81–1.96)
*Alcohol use before sex*	0.99 (0.74–1.32)	1.04 (0.77–1.41)	1.05 (0.81–1.35)	**1.17 (1.01–1.36)**	1.33 (0.92–1.93)	0.83 (0.58–1.19)
*Partner’s alcohol use before sex*	1.25 (0.95–1.64)	0.93 (0.65–1.31)	1.18 (0.93–1.51)	1.15 (0.98–1.33)	0.65 (0.39–1.08)	**1.87 (1.37–2.54)**
*Perceived likelihood of partner being exposed to HIV*						
-Very likely or somewhat likely	1.17 (0.58–2.34)	**2.18 (1.47–3.23)**	**2.37 (1.83–3.06)**	**1.79 (1.30–2.46)**	1.41 (0.71–2.81)	**2.39 (1.51–3.79)**
*Number of partners outside the community: Past 12 months*		-		-	-	
One partner	0.86 (0.60–1.23)		1.22 (0.98–1.52)			
Two or more partners	0.97 (0.65–1.44)		0.92 (0.64–1.32)			
*Partner’s movement*		–			–	
Regular resident of the community	0.95 (0.64–1.42)			–		
Not a regular						
Household characteristics						
*Household wealth*					–	–
High	–	–	–	–		
Middle	1.45 (0.87–2.44)	0.95 (0.78–1.17)		1.07 (0.81–1.41)		1.09 0.80–1.48)
Low	1.28 (0.87–1.88)	1.18 (0.89–1.57)		**1.24 (1.04–1.47)**		0.91 (0.61–1.37)
Family size	**1.07 (1.01–1.14)**	**0.86 (0.81–0.92)**	**0.91 (0.85–0.98)**	**0.94 (0.90–0.97)**	0.95 (0.85–1.07)	**0.89 (0.82–0.96)**

Bold presents significant variables; Only variables significant at .05 level in the univariate analysis were entered into the multivariate model; The types of occupation categories were changed for males and females in fishing, trading and agrarian communities. In fishing communities, fishing was the reference group for males with other occupation categories being agriculture, business and other; In trading communities, males’ occupation types were classified into trading, agriculture and other with trading being the reference group; For males in agrarian communities, the reference group was agriculture, with business/trading and other categories. For women, occupation categories included housework, agriculture, business, hotel/bar and other with housework as the reference group

##### Trading Communities

In trading communities, female gender was significantly related to a positive HIV status. Men were 57% less likely to be HIV positive than women (*adjPRR* = 0.43, *95%* CI = 0.21–0.89). Participants in current or previous marital relationships were more likely to be HIV positive than those who were never married. Belief in partner being HIV positive was positively and significantly associated with HIV positive status (*adjPRR* = 3.13, *95% CI* = 1.31–7.47).

In the stratified analysis of trading communities by gender (Table 3), men in the older age groups were more likely to be HIV positive than those in the younger age groups. Men who were from Catholic religion were more likely to be HIV positive than those from Protestant or Muslim religious background. Among partner variables, both men and women’s current or previous marital status was significantly related to HIV positive status. However, perceptions of partner being HIV positive was significantly related to HIV positive status among men, but not among women.

##### Agrarian Communities

In the overall sample of agrarian communities, being in the older age range (*adjPRR* = 1.65–1.68) or being currently (*adjPRR* = 2.59–3.49) or previously married (*adjPRR* = 4.65, *95% CI* = 2.37–9.11) were significantly related to HIV positive status. Participants who reported initiating sex in childhood (12 years and under; *adjPRR* = 1.65, *95% CI* = 1.02–2.66) or adolescence (13–17 years of age; *adjPRR* = 1.48, *95% CI* = 1.12–1.96) were more likely to be HIV positive than those who initiated sex at 18 years of age or later. Having multiple sexual relationships (*adjPRR* = 1.47, *95% CI* = 1.07–2.01), partner likelihood of being exposed to HIV (*adjPRR* = 2.38, *95% CI* = 1.75–3.24) and small family sizes (*adjPRR* = 0.89, *95% CI* = 0.83–0.96) were also significant factors associated with HIV positive status among agrarian participants.

In the stratified analysis by gender (Table 3), both men and women in the 25–34-year age groups were significantly more likely to be HIV positive than those in 15–24-year age range. Occupation type was significant for women, with those working in bars/restaurants more likely to be HIV positive than those involved in housework. Among relationship factors, while current and previous marital status was associated with HIV positive status among men, only previous marital status was significantly related to HIV positive status among women. Both agrarian men and women in multiple sexual relationships and those who perceived their partners to be HIV positive were significantly more likely to be HIV positive themselves. Partner’s alcohol use was a significant factor for women with women whose partners used alcohol before sex more likely to be HIV positive (*adjPRR* = 1.87, *95% CI* = 1.37–2.54). Among household variables, belonging to a smaller size family was inversely associated with HIV infection among both men (*adjPRR* = 0.91, *95% CI* = 0.85–0.98) and women (*adjPRR* = 0.89, *95%* CI = 0.82–0.96).

#### STI Outcome

##### Fishing Communities

Female gender and being married with multiple partners (*adjPRR* = 2.28, *95% CI* = 1.26–4.13) were factors significantly associated with reporting an STI. Younger age groups in the fishing communities appeared to be more at risk, with those in the age range 25–34 (*adjPRR* = 0.67, *95% CI* = 0.44–0.99) significantly less likely than those in 15–24-year age range to report having an STI.

In the stratified analysis by gender (Table 4), men in fishing communities who always used condoms (*adjPRR* = 0.38, *95% CI* = 0.24–0.63) were significantly less likely to report STI than those who never used condoms. Among women, increased years of education was associated with being less likely to report STI symptoms (*adjPRR* = 0.94, *95% CI* = 0.89–0.99). Physical or sexual IPV in the past year was significantly associated with STI symptoms among women in fishing communities (*adjPRR* = 1.31, *95% CI* = 1.08–1.58).

**Table 4:** STI SYMPTOMS AS OUTCOMES: IPV and Other Factors Related to STI Symptoms among Males and Females in the Three Community types

	Males			Females		
	Fishing	Trading	Agrarian	Fishing	Trading	Agrarian
Individual characteristics						
*Age*						
15 to 24 years (Ref)					–	–
25 to 34 years (2)	1.02 (0.74–1.42)	1.02 (0.56–1.83)	–	–	0.90 (0.75–1.08)	1.16 (0.86–1.57)
35 years and above (3)	0.82 (0.53–1.27)	0.77 (0.40–1.46)			**0.69 (0.52–0.92)**	0.62 (0.32–1.22)
*Marital Status*						
Never married (Ref)	–	–	–	–	–	–
Married with multiple partners (1)	1.19 (0.80–1.78)			1.00 (0.61–1.65)	**1.94 (1.33–2.83)**	0.92 (0.55–1.55)
Married- monogamous (2)	1.15 (0.67–1.98)			1.02 (0.62–1.69)	**1.72 (1.15–2.57)**	1.57 (0.97–2.54)
Previously married (3)	1.24 (0.86–1.79)			1.15 (0.69–1.91)	**1.74 (1.13–2.67)**	1.34 (0.84–2.13)
*Religion*						
Catholic	–	–	–	–	–	–
Protestant	0.89 (0.59–1.34)					
Muslim	0.83 (0.55–1.26)					
Other or None	**1.71 (1.14–2.54)**					
*Education (Years)*		–		**0.94 (0.89–0.99)**	–	–
*Type of Occupation*						
Fishing	Ref	–	–		–	
Housework	–		–		Ref	–
Agriculture	0.62 (0.22–1.70)		Ref	–	1.04 (0.76–1.43)	
Business/Trading	1.32 (0.96–1.82)		1.09 (0.62–1.91)		**1.38 (1.04–1.83)**	
Bar/Restaurant Worker/Waitress/Waiter	–		–		1.08 (0.80–1.46)	
Other	0.73 (0.51–1.03)		1.01 (0.68–1.51)		0.93 (0.71–1.22)	
*Age of first sexual intercourse*						
18 and older (Ref)	–	–	–	–	–	–
Childhood (12 years and under)	0.79 (0.36–1.74)					0.80 (0.40–1.60)
Adolescence (13–17 years)	1.11 (0.87–1.40)					0.99 (0.73–1.33)
*Forced first sex*	–	–	–	1.10 (0.91–1.34)	–	1.27 (0.73–2.19)
Relationship charactersitics						
*Any Physical or Sexual IPV*	1.31 (0.98–1.76)	1.53 (0.93–2.50)	1.29 (0.79–2.11)	**1.31 (1.08–1.58)**	**1.59 (1.37–1.84)**	1.15 (0.88–1.52)
*Age Gap Between Partners*						
1–5 years’ gap (Ref)	–	–	–	–	–	–
6–10 years’ gap (2)						1.00 (0.78–1.29)
11 or more years (3)						1.40 (0.84–2.33)
*Condom use in past year*						
Never used condom (Ref)				–		
Sometimes used condom	0.80 (0.62–1.03)	0.80 (0.62–1.04)	0.95 (0.65–1.39)		–	–
Always used condom	**0.38 (0.24–0.63)**	**0.41 (0.23–0.69)**	0.61 (0.33–1.13)			
*Multiple Sexual Partners (Two or more sexual partners: Past 12 months)*	1.33 (0.89–1.99)	**2.09 (1.21–3.62)**	1.74 (0.93–3.24)	1.14 (0.91–1.42)	**1.50 (1.06–2.10)**	1.36 (0.77–2.41)
*Partner’s multiple sexual relationships*	–	–	–	1.25 (0.96–1.63)	–	**1.44 (1.05–2.00)**
*Alcohol use before sex*	1.12 (0.82–1.52)	1.45 (0.96–2.19)	1.22 (0.76–1.94)	1.10 (0.89–1.35)	1.02 (0.74–1.39)	1.19 (0.86–1.65)
*Partner’s alcohol-use before sex*	1.22 (0.93–1.61)	–	0.95 (0.65–1.39)	1.20 (0.98–1.48)	1.16 (0.94–1.44)	0.98 (0.67–1.44)
*Perceived likelihood of partner being exposed to HIV*						1.09 (0.74–1.60)
-Very likely or somewhat likely	1.31 (0.79–2.17)	1.78 (0.88–3.59)	1.45 (0.80–2.64)	1.22 (0.85–1.76)	1.35 (0.99–1.83)	
*Number of partners outside the community: Past 12 months*		-	-	-	-	-
One partner	0.88 (0.62–1.24)		1.75 (0.87–3.52)	1.10 (0.88–1.37)	0.95 (0.73–1.23)	
Two or more partners	1.31 (0.94–1.81)		1.65 (0.55–4.90)	1.25 (0.74–2.14)	1.15 (0.70–1.89)	
*Partner’s movement*						
Regular resident of the community (Ref)	–	–	–	–	–	–
Not a regular	0.56 (0.31–1.02)		0.72 (0.48–1.07)			
Household characteristics						
*Family size*	0.97 (0.92–1.02)	0.95 (0.91–1.00)	**0.86 (0.79–0.94)**	0.97 (0.92–1.02)	**0.91 (0.86–0.95)**	**0.92 (0.85–0.98)**

Bold presents significant variables; Only variables significant at .05 level in the univariate analysis were entered into the multivariate model; The types of occupation categories were changed for males and females in fishing, trading and agrarian communities. In fishing communities, fishing was the reference group for males with other occupation categories being agriculture, business and other; In trading communities, males’ occupation types were classified into trading, agriculture and other with trading being the reference group; For males in agrarian communities, the reference group was agriculture, with business/trading and other categories. For women, occupation categories included housework, agriculture, business, hotel/bar and other with housework as the reference group

##### Trading Communities

Always using condoms was a protective factor for STI among men in trading communities. However, engagement in multiple sexual relationships (*adjPRR* = 2.09, *95% CI* = 1.21–3.62) was correlated with self-reported STI symptoms among men. Engagement in multiple sexual relationships was also related to STI symptoms among women (*adjPRR* = 1.50, *95% CI* = 1.06–2.10). Other correlates of STIs among women in trading communities included being in current or previous marital relationships, type of occupation (i.e., engagement in business or trading occupations versus housework (*adjPRR* = 1.38, *95% CI* = 1.04–1.83), and exposure to physical or sexual violence (*adjPRR* = 1.59, *95% CI* = 1.37–1.84; Table 4).

##### Agrarian Communities

In the total sample of agrarian communities, agrarian women and those in the younger age groups appeared to be at greater risk for STI symptoms. Participants in the 35 and above age category were significantly less likely to have an STI than those in 15–24 age range (*adjPRR* = 0.53, *95% CI* = 0.36–0.78). Participants in current monogamous marital relationships were significantly more likely to report an STI than those who were never married (*adjPRR* = 5.14, *95% CI* = 1.79–14.7). Multiple sexual relationships (*adjPRR* = 1.48, *95% CI* = 1.04–2.09) were also significantly related to STI symptoms. Participants belonging to larger families were 9% less likely than those from smaller families to report an STI (*adjPRR* = 0.91, *95% CI* = 0.85–0.97).

In stratified analysis, both men and women from smaller families were more likely to report STI symptoms than those from larger families. Other factors were not related to STIs among agrarian men. In contrast, having a partner with multiple sexual relationships appeared to be correlated with self-reported STI among agrarian men (*adjPRR* = 1.44, *95% CI* = 1.05–2.00; Table 4).

#### IPV Outcome

##### Fishing Communities

Women in fishing communities were more likely to have reported IPV than men. Men and women who provided any one justification for IPV were more likely to report recent IPV perpetration or victimization, respectively (*adjPRR* = 3.26, *95% CI* = 1.74–6.10). Those who always used condoms were significantly less likely than those who never used condoms to have reported IPV (*adjPRR* = 0.45, *95% CI* = 0.25–0.81). Perception of partner being HIV positive was significantly associated with IPV in fishing communities (*adjPRR* = 1.72, *CI* = 1.02–2.90).

In stratified analysis by gender (Table 5), factors such as being married with monogamous marital status (*adjPRR* = 1.73, *95% CI* = 1.02–2.93), having multiple sex partners (*adjPRR* = 2.32, *95% CI* = 1.11–4.85) and having any justification for violence against women (*adjPRR* = 3.05, *95% CI* = 1.99–4.69) were significantly associated with recent perpetration of IPV by men. Men who always used condoms were significantly less likely than those who never used condoms to have perpetrated violence against their female partner (*adjPRR* = 0.45, *95% CI* = 0.28–0.72). Among women in fishing communities, those who were younger, HIV positive (*adjPRR* = 1.28, *95% CI* = 1.00–1.65), reported a recent STI (*adjPRR* = 1.37, *95% CI* = 1.07–1.76), had multiple sex partners (*adjPRR* = 1.39, *95% CI* = 1.05–1.83) and had a partner who used alcohol before sex (*adjPRR* = 1.42, *95% CI* = 1.10–1.83), were significantly more likely to have experienced IPV than other women.

**Table 5:** Intimate partner violence as an outcome: factors related to IPV among males and females in the three community types

	Males			Females		
	Fishing	Trading	Agrarian	Fishing	Trading	Agrarian
	IPV Perpetration			IPV Victimization		
Individual characteristics						
*Age*						
15 to 24 years (Ref)	–				–	–
25 to 34 years (2)	0.83 (0.57–1.21)	–	–	0.82 (0.61–1.09)		
35 years and above (3)	0.67 (0.40–1.12)			**0.65 (0.43–0.98)**		
*Marital Status*						
Never married (Ref)	–	–	–	–	–	
Married with multiple partners (1)	1.51 (0.87–2.59)	1.31 (0.82–2.08)	1.95 (0.97–3.92)	0.82 (0.46–1.44)	1.13 (0.49–2.58)	–
Married- monogamous (2)	**1.73 (1.02–2.93)**	1.29 (0.68–2.42)	**2.16 (1.16–4.03)**	0.91 (0.52–1.57)	**2.37 (1.08–5.21)**	
Previously married (3)	1.39 (0.82–2.36)	0.81 (0.29–2.27)	1.07 (0.40–2.83)	0.63 (0.35–1.12)	0.59 (0.22–1.59)	
*Children*						
***0–1*** child			–	–	–	–
2–3 children				1.11 (0.82–1.49)		
4–5 children				1.07 (0.71–1.60)		
6 children and above				1.11 (0.66–1.89)		
*Religion*						
Catholic	–	–	–	–	–	
Protestant	**0.49 (0.26–0.92)**				0.78 (0.49–1.22)	
Muslim	0.95 (0.64–1.40)				0.54 (0.27–1.09)	
Other or None	0.73 (0.32–1.67)				0.78 (0.23–2.58)	
*Education (Years)*		–	–	–	–	–
*Type of Occupation*						
Fishing	–	–		–	–	
Housework		–		Ref	Ref	
Agriculture				0.87 (0.51–1.48)	1.03 (0.51–2.07)	–
Business/Trading				0.81 (0.56–1.16)	1.34 (0.65–2.75)	
Bar/Restaurant				1.11 (0.79–1.57)	1.35 (0.51–3.61)	
Other				0.91 (0.65–1.29)	0.68 (0.30–1.51)	
*Age of first sexual intercourse*						
18 and older (Ref)	–	–	–	–	–	–
Childhood (12 years and under)	1.53 (0.88–2.65)				1.58 (0.85–2.93)	1.00 (0.32–3.06)
Adolescence (13–17 years)	0.97 (0.73–1.30)				1.15 (0.72–1.84)	0.52 (0.23–1.19)
*Forced first sex*	–	–	–	1.12 (0.87–1.45)	1.05 (0.63–1.76)	1.83 (0.67–5.05)
*HIV Status*	1.08 (0.82–1.43)		1.05 (0.65–1.68)	**1.28 (1.00–1.65)**	1.26 (0.78–2.03)	–
*STI Symptoms*	1.24 (0.93–1.65)	1.72 (0.97–3.03)	1.23 (0.81–1.87)	**1.37 (1.07–1.76)**	**1.45 (1.01–2.09)**	2.03 (0.91–4.52)
Relationship charactersitics						
*Age Gap Between Partners*						
1–5 years’ gap (Ref)	–					
6–10 years’ gap (2)	0.75 (0.50–1.14)	–	–			–
11 or more years (3)	1.20 (0.68–2.13)			–	–	
*Condom use in past year*						
Never used condom (Ref)	–	–	–			–
Sometimes used condom	0.93 (0.69–1.31)	0.75 (0.52–1.09)	1.20 (0.72–1.97)	–	–	
Always used condom	**0.45 (0.28–0.72)**	0.42 (0.17–1.04)	0.71 (0.39–1.27)			
*Multiple sexual partners: Past 12 months*	**2.32 (1.11–4.85)**	**4.57 (1.87–11.1)**	1.92 (0.83–4.44)	**1.39 (1.05–1.83)**	1.06 (0.65–1.71)	**5.71 (2.56–12.71)**
*Partner’s multiple sexual relationships*	–	–	–	1.20 (0.89–1.62)	**3.54 (1.94–6.48)**	1.51 (0.56–4.01)
*Recent casual relationship*						
-A spouse/consensual regular partner (Ref) -Irregular partner	- 1.10 (0.69–1.75)	–	–	–	–	–
*Alcohol use before sex*	1.13 (0.81–1.57)	1.62 (0.99–2.64)	1.44 (0.88–2.36)	0.96 (0.74–1.25)	1.13 (0.78–1.63)	0.94 (0.31–2.86)
*Partner’s alcohol use before sex*	0.95 (0.69–1.31)	1.46 (0.76–2.79)	**1.56 (1.03–2.35)**	**1.42 (1.10–1.83)**	**1.53 (1.01–2.33)**	2.27 (0.71–7.27)
*Perceived likelihood of partner being exposed to HIV*			-			0.72 (0.24–2.11)
-Very likely or somewhat likely				1.33 (0.84–2.11)	1.24 (0.72–2.13)	
*Number of partners outside the community: Past 12 months*	-	-	-	-	-	-
One partner			1.30 (0.72–2.36)	0.85 (0.61–1.19)	1.18 (0.79–1.78)	
Two or more partners			**1.94 (1.05–3.60)**	1.28 (0.81–2.01)	**2.86 (1.09–7.47)**	
Household characteristics						
*Household wealth*			–		–	–
High	–	–		–		**2.26 (1.00–5.11)**
Middle						0.94 (0.27–3.18)
Low						
*Family size*	1.02 (0.96–1.09)	0.93 (0.83–1.04)	0.98 (0.91–1.07)	0.96 (0.89–1.04)	1.07 (0.99–1.14)	1.08 (0.99–1.19)
Social/community norms						
*Any Justification of wife/partner beating*	**3.05 (1.99–4.69)**	–	1.49 (0.86–2.57)	1.38 (0.98–1.95)	–	1.69 (0.85–3.38)

Bold presents significant variables; Only variables significant at .05 level in the univariate analysis were entered into the multivariate model; The types of occupation categories were changed for males and females in fishing, trading and agrarian communities. In fishing communities, fishing was the reference group for males with other occupation categories being agriculture, business and other; In trading communities, males’ occupation types were classified into trading, agriculture and other with trading being the reference group; For males in agrarian communities, the reference group was agriculture, with business/trading and other categories. For women, occupation categories included housework, agriculture, business, hotel/bar and other with housework as the reference group

##### Trading communities

In gender-stratified analysis (Table 5), having multiple sex partners was significantly associated with men’s perpetration of IPV (*adjPRR* = 4.57, *95% CI* = 1.87–11.1). Women in trading communities who were married with monogamous marital status (*adjPRR* = 2.37, *95% CI* = 1.08–5.21) but had a partner with multiple sexual relationships (*adjPRR* = 3.54, *95% CI* = 1.94–6.48), had two or more partners outside the trading community (*adjPRR* = 2.86, *95% CI* = 1.09–7.47) and had an STI (*adjPRR* = 1.45, *95% CI* = 1.01–2.09) were significantly more likely to be victimized by IPV than other women.

##### Agrarian communities

In agrarian communities, men in monogamous marriages (*adjPRR* = 2.16, *95% CI* = 1.16–4.03), but had two or more partners outside the community (*adjPRR* = 1.94, *95% CI* = 1.05–3.60) and had partners who used alcohol before sex (*adjPRR* = 1.56, *95% CI* = 1.03–2.35) were more likely to report perpetration of IPV. In contrast, agrarian women from middle income households (*adjPRR* = 2.26, *95% CI* = 1.00–5.11) and those with multiple sex partners (*adjPRR* = 5.71, *95% CI* = 2.56–12.7) were more likely than others to have been victimized by IPV (Table 5).

## Discussion

We found significantly higher prevalence of HIV infection and past year self-reports of IPV victimization and perpetration, and STI symptoms in fishing communities, compared to trading and agrarian communities in Rakai, Uganda. Syndemic effects of all outcomes (HIV infection, IPV and STI symptoms) were found among women: past year IPV victimization was significantly associated with HIV positive status and self-reported STI symptoms during the same time frame in the fishing communities. Multiple syndemic mechanisms offer explanation to the pronounced links between HIV risk and infection, STI symptomology and experience of violence among women in Rakai’s fishing communities. At the relationship level, women in violent relationships commonly lack agency to negotiate safe sex practices with their partners and if they do suggest condom use or lack of interest in sexual intimacy, this may result in forced sex [26]. Abusive partners are more likely than non-abusive partners to have multiple sexual relationships and engage in forced unprotected sex with their partners, placing them at risk for HIV/STI [27]. This is supported by our finding on the association of IPV perpetration with inconsistent condom use among men in fishing communities. Biologically, IPV can negatively impact immune system functioning, thus reducing immunity to HIV acquisition and leading to increased disease progression in HIV-infected women [7, 21]. In terms of health systems, limited coverage of health prevention and treatment services in fishing communities may explain some of the disproportionate burden of HIV infection, STI symptoms and IPV victimization and perpetration found there [25]. This calls for enhanced IPV prevention efforts targeting men in fishing communities in Rakai.

Relative to trade and agrarian areas, a greater proportion of participants from fishing communities reported HIV transmission risk behaviors in the past year, including multiple sex partners, casual relationships, having a partner with multiple sexual relationships, inconsistent condom use, use of alcohol before sex and partner’s use of alcohol before sex. Consistent with prior research, these behavioral patterns were more common among men than women [25]. Men’s behaviors are likely to have played a role in the presence of syndemic conditions among women in fishing communities. Further, we found that more fishing community participants were from low income households when compared to the other two community types, corroborating findings from other studies in Africa that have associated these risk behaviors and low income with HIV prevalence [19, 28].

Female gender was a shared correlate for the syndemic relationship between HIV infection, STI symptoms and IPV in the fishing communities. Alcohol use, however, was only a common correlate of IPV and HIV infection among fishing communities’ participants. Multiple sexual relationships were significantly associated with IPV perpetration (men) and victimization (women) in fishing, trading and agrarian communities. Multiple sexual relationships or partner’s multiple sexual relationships were shared correlates for IPV and STI in trading communities and IPV and HIV in agrarian communities. Research from African settings show that levels of IPV are much higher among women with multiple sexual partners than women with only one partner [29]. In our sample, we could not explore the reasons for the relationship between multiple sex partners and IPV since that data was not collected. In research by Zembe and colleagues (2015), findings suggested that sex in exchange of money and/or gifts boosts male power and sexual entitlement, leading to IPV in cases where women tried to evade sex [29].

Social norms play an important role in IPV victimization and justification. Any justification of physical violence against a partner appear to increase risk for IPV perpetration among men in the fishing community. Social norms that allow and condone violence against women and norms related to safe sex practices must be considered in developing appropriate strategies for IPV and HIV/STI prevention in the fishing community. Promoting gender-egalitarian norms and safe sex norms can lead to behavior change and prevent risk for IPV, HIV and STI among couples in fishing communities.

Partner’s use of alcohol before sex was a significant risk factor for IPV victimization among women in fishing and trading communities, and IPV perpetration among men in agrarian communities. Problematic alcohol use has been associated with IPV perpetration as well as HIV infection in studies in Africa [22, 30]. In our study, women’s use of alcohol before sex in fishing communities and partner’s use of alcohol before sex in agrarian communities were significantly related to HIV infection among women. Thus, alcohol use can place individuals at risk for both IPV and HIV. IPV and HIV prevention efforts in Uganda, therefore, need to address alcohol use among women as well as their partners. RHSP adapted, implemented and evaluated multiple community and clinic level approaches to address and reduce the intersecting epidemics of IPV and HIV infection, as part of what was named the Safe Homes and Respect for Everyone (SHARE) Project. SHARE was delivered and evaluated between 2005 and 2009 and found to be associated with significant decreases in physical and sexual IPV (including forced sex) against women and HIV incidence in the general population [31, 32]. RHSP’s IPV and HIV prevention efforts (i.e., the SHARE intervention), however, were limited to only 4 of the 11 Rakai’s trading and agrarian communities. Since most of the agrarian and trading communities were not exposed to SHARE, the two communities are unlikely to differ from fishing communities in IPV rates due to non-exposure to SHARE.

Beyond interpersonal risks, the contexts within which people live also plays an important role in HIV/STI acquisition and IPV. For instance, RHSP data from local fishing communities highlight significant disparities in HIV prevalence and incidence. In 2011, HIV prevalence in the fishing communities was estimated at 42.4% with high incidence of 3.9/100 person years where, by comparison, HIV prevalence was 17% in trading and 14% in agrarian communities [25]. The magnitude of IPV, however, as well as its associations with HIV infection have not been established in Rakai’s fishing areas. These findings can be used to further expand SHARE to address the syndemics of IPV, HIV and STI in the fishing communities in Rakai.

In fishing communities, women from low income households were more likely to be HIV infected than other women. Studies have found a relationship between low socio-economic status and HIV prevalence [20, 31]. Women who come from low income households may engage in HIV risk behaviors such as transactional sex for money. Further, a low-income background reduces access to information about safe sex practices and inhibits ability to put this knowledge into practice [31]. We found education to be protective against STIs among women in fishing communities. In a systematic review in Africa, HIV prevalence was found to decrease consistently over the years (1987–2003) among highly educated groups [32]. Thus, programs are needed to promote education, particularly among low-income households in Uganda such as PREPARE, a school-based HIV and IPV prevention program on adolescent sexual risk behavior and IPV [33].

Belonging to a large family was a protective factor for HIV and STI among women in fishing, trading and agrarian communities. A large family can be a source of social support. In a systematic review, existing studies indicated that higher levels of social support, consistent with HIV prevention efforts, are related to fewer HIV-related risk behaviors among heterosexual adults in general and female sex workers [34]. In contrast, with the other groups, we found that men in the fishing communities with larger families were more likely to be HIV infected than those with smaller families. One explanation could be the characteristics or social norms of the social support men in fishing communities receive from their families. Social norms (e.g., norms related to condom use) being inconsistent with HIV prevention may reinforce HIV risk behaviors [34]. HIV prevention efforts for men in fishing communities must consider dynamics of relationships within the family and other people in their social networks.

The strengths of this study include a large sample size and identification of shared or unique factors related to HIV infection, STI symptoms and IPV outcomes comparing the three communities and incorporating gender differences. Limitations include the use of self-reported measures for most variables including STI symptoms and IPV. Self-reported STI symptomatology can serve as a proxy for STI infection but might not be fully accurate and could either over or underestimate the magnitude of problems. For IPV, the measures were dichotomized and did not capture frequency or severity of IPV. Future research may include biological measures for STI and more in-depth examination of characteristics of IPV. In addition, we conducted a cross-sectional analysis and were unable to determine the direction of causality. Despite these limitations, the study provides useful insights on differences between the three communities and gender differences within each community on factors related to HIV infection, STI symptoms and IPV victimization and perpetration. This research supports the need for contextually integrated and gender specific integrated HIV/STI and IPV prevention and interventions for the residents of fishing, trading and agrarian communities in Rakai, Uganda.

## Conclusion

Using a syndemic perspective to understand intersecting epidemics of HIV, STI and IPV and identify shared risk factors that influence HIV, STI and IPV can inform public health interventions among populations in Rakai, particularly those in fishing communites. Our findings can be useful for developing targeted combination interventions for fishing communities in Rakai, Uganda. Interventions may address risky behaviors among the high-risk groups and norms supporting use of violence against women. Our research also suggests that men and women may have different HIV, STI and IPV prevention needs. This calls for gender-focused approaches to address factors that contribute to HIV, STI and IPV.

## Abbreviations

adjPRR: adjusted prevalence ratios
CI: Confidence Interval
EIA: Enzyme Immunoassays
HIV: Human Immunodeficiency Virus
IPV: Intimate partner violence
RCCS: Rakai Community Cohort Study
STI: Sexually Transmitted Infections

## References

[1] Global and regional estimates of violence against women: prevalence and health effects of intimate partner violence and non-partner sexual violence. Available from: https://apps.who.int/iris/bitstream/handle/10665/85239/9789241564625_eng.pdf;jsessionid=5370F847991660F291CE61D59ECAC2EE?sequence=1. Accessed 15 Jan 2018.

[2] UNAIDS. Global AIDS Update 2016. Available from: http://www.unaids.org/sites/default/files/media_asset/global-AIDS-update-2016_en.pdf. Accessed 15 Jan 2018.

[3] Callands TA, Sipsma HL, Betancourt TS, Hansen NB (2013). Experiences and acceptance of intimate partner violence: associations with sexually transmitted infection symptoms and ability to negotiate sexual safety among young Liberian women. Cult health sex.

[4] Wagman JA, King EJ, Namatovu F, Kiwanuka D, Kairania R, Semanda JB (2016). Combined intimate partner violence and HIV/AIDS prevention in rural Uganda: design of the SHARE intervention strategy. Health Care Women Intl.

[5] Kouyoumdjian FG, Calzavara LM, Bondy SJ (2013). Intimate partner violence is associated with incident HIV infection in women in Uganda. AIDS.

[6] Li Y, Marshall CM, Rees HC, Nunez A, Ezeanolue EE, Ehiri JE (2014). Intimate partner violence and HIV infection among women: a systematic review and meta-analysis. J Int AIDS Soc.

[7] Mitchell J, Wight M, Van Heerden A, Rochat TJ (2016). Intimate partner violence, HIV, and mental health: a triple epidemic of global proportions. Int Rev Psychiatry.

[8] Stockman JK, Lucea MB, Draughon JE (2013). Intimate partner violence and HIV risk factors among African-American and African-Caribbean women in clinic-based settings. AIDS care.

[9] Campbell JC, Baty ML, Ghandour RM, Stockman JK, Francisco L, Wagman J (2008). The intersection of intimate partner violence against women and HIV/AIDS: a review. Int J Inj Control Saf Promot.

[10] Zembe YZ, Townsend L, Thorson A, Silberschmidt M, Ekstrom AM (2015). Intimate partner violence, relationship power inequity and the role of sexual and social risk factors in the production of violence among young women who have multiple sexual partners in a peri-urban setting in South Africa. PLoS One.

[11] Mumtaz GR, Weiss HA, Thomas SL (2014). HIV among people who inject drugs in the Middle East and North Africa: systematic review and data synthesis. PLoS Med.

[12] Koenig M, Zablotska M, Lutalo T, Nalugoda F, Wagman J, Gray R (2004). Coerced first intercourse and reproductive health among adolescent women in Rakai, Uganda. Int Fam Plan Perspect.

[13] Singer M, Clair S (2003). Syndemics and public health: Reconceptualizing disease in bio-social context. Med Anthropol Q.

[14] Singer MC, Erickson PI, Badiane L, Diaz R, Ortiz D, Abraham T, Nicolaysen AM (2006). Syndemics, sex and the city: understanding sexually transmitted diseases in social and cultural context. Soc Sci Med.

[15] Polis CB, Lutalo T, Wawer M (2009). Coerced sexual debut and lifetime abortion attempts among women in Rakai, Uganda. Int J Gynecol Obstet.

[16] Wagman JA, Charvat B, Thoma ME (2016). Intimate partner violence as a predictor of marital disruption in rural Rakai, Uganda: a longitudinal study. Int J Public Health.

[17] Audet CM, Burlison J, Moon TD, Sidat M, Vergara AE, Vermund SH (2010). Sociocultural and epidemiological aspects of HIV/AIDS in Mozambique. BMC Int Health Hum Rights.

[18] Papworth E, Ceesay N, An L (2013). Epidemiology of HIV among female sex workers, their clients, men who have sex with men and people who inject drugs in west and Central Africa. J Int AIDS Soc.

[19] Rosenthal L, Scott DP, Kelleta Z (2003). Assessing the HIV/AIDS health services needs of African immigrants to Houston. AIDS Educ Prev.

[20] Scorgie F, Chersich MF, Ntaganira I, Gerbase A, Lule F, Lo Y-R (2012). Socio-demographic characteristics and behavioral risk factors of female sex workers in sub-Saharan Africa: a systematic review. AIDS Behav.

[21] Edwards KM (2015). Intimate partner violence and the rural–urban–suburban divide. Trauma violence Abuse.

[22] Iyoke C, Ajah L, Nkwo P, Nwakoby B, Ezeonu P (2014). Comparison of domestic violence against women in urban versus rural areas of Southeast Nigeria. Int J Womens Health.

[23] Wawer MJ, Gray RH, Sewankambo NK, Serwadda D, Paxton L, Berkley S, Quinn TC (1998). A randomized community trial of intensive sexually transmitted disease control for AIDS prevention, Rakai, Uganda. AIDS.

[24] Straus MA (1979). Measuring Intrafamily conflict and violence: the conflict tactics (CT) scales. J Marriage Fam.

[25] Chang LW, Grabowski MK, Ssekubugu R (2016). Heterogeneity of the HIV epidemic in agrarian, trading, and fishing communities in Rakai, Uganda: an observational epidemiological study. Lancet HIV.

[26] Wirtz AL, Schwartz S, Ketende S, Grosso A, Anato S, Djarki N (2015). Sexual violence and condom use in the context of sex work: results from two West African countries. J Acquir Immune Defic Syndr.

[27] Coker AL (2007). Does physical intimate partner violence affect sexual health? A systematic review. Trauma Violence Abuse.

[28] Townsend L, Jewkes R, Mathews C (2011). HIV risk behaviours and their relationship to intimate partner violence (IPV) among men who have multiple female sexual partners in Cape Town, South Africa. AIDS Behav.

[29] Wagman JA, Namatovu F, Nalugoda F (2012). A public health approach to intimate partner violence prevention in Uganda. Violence against women.

[30] Wagman JA, Gray RH, Campbell JC (2015). Effectiveness of an integrated intimate partner violence and HIV prevention intervention in Rakai, Uganda: analysis of an intervention in an existing cluster randomised cohort. Lancet glob heal.

[31] Hallman K (2005). Gendered socioeconomic conditions and HIV risk behaviours among young people in South Africa. Afr J AIDS Res.

[32] Hargreaves JR, Bonell CP, Boler T (2008). Systematic review exploring time trends in the association between educational attainment and risk of HIV infection in sub-saharan Africa. Aids.

[33] Mathews C, Eggers SM, Townsend L, Aaro LE, de Vries PJ, Mason-Jones AJ (2016). Effects of PREPARE, a multicomponent, school-based HIV and intimate partner violence (IPV) prevention programme on adolescent sexual risk behavior and IPV: cluster randomized controlled trial. AIDS Behav.

[34] Qiao S, Li X, Stanton B (2014). Social support and HIV-related risk behaviors: a systematic review of the global literature. AIDS Behav.

